# Companion animal adoption and relinquishment during the COVID-19 pandemic: Peri-pandemic pets at greatest risk of relinquishment

**DOI:** 10.3389/fvets.2022.1017954

**Published:** 2022-09-30

**Authors:** Grace A. Carroll, Alice Torjussen, Catherine Reeve

**Affiliations:** ^1^Animal Behaviour Centre, School of Psychology, Queens University Belfast, Belfast, United Kingdom; ^2^Animal-Computer Interaction Lab, School of Engineering and Informatics, University of Sussex, Falmer, United Kingdom

**Keywords:** adoption, relinquishment, cat, dog, COVID-19, online

## Abstract

The COVID-19 pandemic has created a situation globally where companion animals may be at increased risk of relinquishment and abandonment due to multiple interrelated factors. The aims of this study were to establish the prevalence of self-reported adoption and relinquishment of cats and dogs during the pandemic, and to identify characteristics associated with relinquishment. A survey was distributed to 4,000 participants across several countries including the UK, USA, Canada, Italy, Spain and France. *N* = 3,945 responses were available for analysis. Three groups of participants were identified; Those that never considered relinquishment (NCR), those that have considered relinquishment (CR) and those that have already relinquished a cat or dog (R). Two follow-up surveys were sent to CR and R participants. Considering data from the three surveys, 4.06% of participants considered giving up their pet, 0.74% relinquished their pet, and 0.2% considered and then later give up their pet. Compared to pets given as a gift, there was a 38.7% decreased likelihood of relinquishment in pets sourced from a shelter (*P* < 0.001), 31.2% decrease in those sourced from a breeder, and a 24.4% decrease in those acquired directly from someone that needed to find a new home for their cat or dog. Compared to owners who acquired their pet > 6 months before COVID-19 was declared a pandemic, those acquired < 6 months before COVID-19 was declared a pandemic were three times more likely to be considered for, or be, given up (*P* < 0.001) and those acquired after COVID-19 was declared a pandemic were two times more likely to be considered for, or be, given up (*P* < 0.001). There was a trend for greater likelihood of CR or R of pets acquired online (*P* = 0.074). Of those that had already given up their pet, 14.3% relinquished to a shelter, 66.7% gave their pet to a new owner and 19% obtained temporary care from someone else. A total of 65.0% of CR participants were male, increasing to 72.2% of R participants. There was no effect of species (cat or dog) on risk of relinquishment. Financial constraints were the most mentioned reason for both giving up a pet and considering giving up a pet, followed by health concerns specific to COVID-19, and behavioral problems. The findings from this study should be used to inform interventions aimed at reducing companion animal relinquishment.

## 1. Introduction

During times of crisis, there is an increased risk to animal welfare (1). However, most of the literature on human-animal relations during crisis situations has focused on natural disasters rather than disease outbreaks (2). The COVID-19 pandemic has created a situation globally where companion animals may be at increased risk of relinquishment and abandonment due to multiple interrelated factors. For example, many citizens have experienced an abrupt loss of income (3) or have begun to work at home where pets may interrupt the working day (4). Similarly, frontline workers face increased workloads and time pressures, leaving less time available to care for pets (5, 6). In times of stress and financial difficulty such as this, animal charities see increased pressure on their veterinary services and an increase in cases of cruelty, neglect and abandonment (7). At the same time, in line with government guidance, animal charities are restricting or suspending adoptions and new animal admissions and veterinarians have been forced to provide restricted services (8–11). Companion animal abandonments and relinquishments may be further increased by fears that COVID-19 can be passed on from companion animals to humans (12).

Given this unique set of circumstances, it is important to establish the impact of the COVID-19 on companion animals by establishing the prevalence of adoption and relinquishment during the pandemic and identifying risk factors for relinquishment. Furthermore, reasons for relinquishment should be assessed as they may vary from those given under normal circumstances. A distinction should be made between relinquishment, abandonment, surrender and transfer, which all refer to giving up a pet, but in different circumstances. According to Sharkin and Ruff (13), relinquishment is where a pet owner voluntarily gives up their companion animal to a shelter; transfer is where pets are given to family members or friends, surrender is where owners are required to give up their pet on an involuntary basis, and abandonment is where companion animals are left without care or any intention of resuming care. For our purposes, the term relinquishment will be used to refer to all manners in which individuals give up a pet (14, 15), unless otherwise specified.

The decision to relinquish a companion animal can take weeks and months of consideration, with pet owners trying to find an alternative home themselves before approaching an animal shelter (13, 16). In addition, Dolan et al. (16) found that when approached outside animal shelters and informed about support services, 88% of people were willing to consider alternatives to relinquishment and left the shelter with their companion animals. Therefore, it may be possible to reduce the number of relinquishments and abandonments by offering owners an alternative.

The circumstances under which a pet is acquired may influence later relinquishment decisions. Currently there is a lack of research into the association between the source of pet acquisition and the ultimate outcome for these animals (17). In particular, there is a lack of research on relinquishment outcomes for animals sourced online, and online pet acquisition more generally (18). Purchasing a pet online is of concern to animal welfare scientists and animal charities alike as they can facilitate impulsive pet acquisition and illegal puppy trading (19, 20). Time of pet acquisition may also influence risk of relinquishment. In general, newly acquired pets are more likely to be relinquished. For example, Shore et al. (21) found that 47.4% of animals relinquished to Midwestern USA animal shelters were in the home for less than a year. Similarly, Shore (22) found that 54% of returned adopted pets were sent back to the shelter within 2 weeks of acquisition, and a further 32% of pets were relinquished between 2 months to a year after acquisition. Only 7% were returned over a year after being adopted. More recently, Powell et al. (10) found that of all adopted animals from one USA shelter, 9.2% were returned within 6-months of adoption. This suggests that the majority of individuals that return pets to shelters do so either in the immediate days following acquisition or after 2 months to a year, perhaps when problems have had time to manifest themselves. There has been an increased adoption of pets during the pandemic, including an increase in impulse buying (23, 24). Consequently, there are fears that an increase in relinquishment will be seen as life returns to normal (25).

Another important influence on relinquishment risk is owner sex and gender. Recent studies that surveyed companion animal owners during the COVID-19 pandemic had a largely female participant base (26, 27). For example, Packer et al. (28) assessed puppy acquisition pre- and post-pandemic, with 90–92% of responses coming from female pet owners. The remaining 8–10% of participants identified as “male”, “other”, or “prefer not to say”. Similarly, Christley et al. (6) assessed the management of pet dogs during the first UK COVID-19 lockdown with 85.7% of participants identifying as female, 14.2% identifying as male, and 0.1% identifying as “other.” Christley et al. (6) highlight the need to recruit a larger sample of males to reduce sampling bias in animal behavior and welfare research. While there is evidence that the male sex relinquish pets more than the female sex (16, 29), males remain under-represented in relinquishment studies, making it difficult to arrive at a definitive conclusion.

This paper is one in a series of publications part of a larger project, “CAARP” (Companion Animal Adoption and Relinquishment during the Pandemic), which seeks to understand adoption and relinquishment of cats and dogs across several countries from the perspective of pet owners, shelter staff, and from shelter records, employing a mixture of qualitative and quantitative approaches to data collection.

The aims of this study were to:

a) Establish the prevalence of self-reported relinquishment of cats and dogs during the COVID-19 pandemic, including pet retention rates over time.
b) Identify acquisition characteristics associated with relinquishment of cats and dogs during the COVID-19 pandemic.
c) Assess the effect of gender on self-reported relinquishment of cats and dogs during the COVID-19 pandemic.
d) Identify the reasons given by companion animal owners for abandonment, relinquishment and transfer of cats and dogs during the COVID-19 pandemic.

## 2. Methods

### 2.1. Study design and participants

A cross-sectional study design was used to assess the prevalence of self-reported relinquishment of cats and dogs during the COVID-19 pandemic *via* an online survey. A sub-set of participants were invited to complete two follow-up questionnaires. Participants were recruited *via* Prolific Academic, an online recruitment site that pays participants to take part in research. Prolific Academic has been shown to provide high quality, reliable data, and has a diverse participant pool (30). A purposive sampling method was employed by utilizing Prolific Academic's participant screening tool. Of the 4,000 study places, 2000 places were allocated to males and 2,000 places were allocated to females. It is important to note that the Prolific Academic pre-screening tool filters respondents by male and female only by using the question: ‘What sex were you assigned at birth, such as on an original birth certificate?'. Participants answer this question with one of three options: male, female, or rather not say. Therefore, when using this pre-screening tool, it is only possible to balance the study according to “male” and “female.” The pre-screening tool was necessary to ensure an even split within the initial survey as we aimed to avoid the issue of female response bias often seen in survey-type research on similar topics, and in survey-type research more generally. In surveys 2 and 3, which focused on those that relinquished their pet, we ensured that participants could specify their gender. Participants were also required to be current or past pet-owners. Participants from several countries were surveyed including the UK, Ireland, Italy, Spain, France, USA, Canada and Australia, with a small number of respondents from other countries. This allowed us to explore the effects of the pandemic in countries at different stages of the pandemic. All data was collected anonymously, with each participant having a unique Prolific ID that allowed participant's responses to be matched across surveys.

### 2.2. Procedure

After screening participants on the Prolific Academic database, the available pool of potential participants was *N* = 31,952. From this pool, participants meeting the screening criteria could complete the study until *n* = 2,000 female and *n* = 2,000 male participants had completed it successfully. Survey 1 was completed on the 11th of August, 2020. Participants were first directed to a participant information sheet and completed a consent form. Participants were instructed to answer the questions with their cat or dog in mind. If they had multiple cats and/or dogs, participants were instructed to answer the questions for the pet that they most recently acquired. The initial survey was comprised of 14 questions including country of residence, animal species (cat or dog), source of acquisition, current ownership status, and whether the participant had ever considered giving up their pet. Those reporting that they had considered, or already had given up a pet, were asked about their reasons for this. After removal of partial and duplicate responses, a total of 3,945 responses were available for analysis. Participants were invited to give more detail on their experiences *via* free text responses for several questions. A sample of these responses are provided throughout the results section. A second survey was distributed 1 week later and was completed between 17th to 24th of August 2020, by those that had reported that they had considered, or had already given up a cat or dog, with *n* = 181 usable responses. Seven months later, a third survey was sent to those that have considered giving up a pet to see if there had been any changes in ownership status during this time period. This was completed between 31st March and 8th April 2021 by *n* = 64 participants. Survey 2 and 3 contained four generic questions, 24 questions for those considering giving up a pet, and 32 questions for those that have already given up their cat or dog. The detailed results from survey 2 and 3 are reported elsewhere (Carroll et al. in preparation). For the purposes of this paper, the number of participants moving from consideration of relinquishment to actual relinquishment over the 7-month period was assessed. At each data collection point, participants verified that they had completed the survey in its entirety by providing a code which was available only on completion. Participants were then paid for their participation at an average rate of £6.50 per hour across the three surveys.

### 2.3. Statistical analysis

Descriptive statistics were used to analyze the demographic information collected for survey 1. A 2 x 2 Pearson's chi-square test was used to explore the relationship between categorical variables “Acquired online” (binary: Yes/No) and “Species” (binary: cat/dog). A 2 x 3 Pearson's chi-square test was used to explore the relationship between “Acquired online” (binary: Yes/No) and “When acquired” (three categories: >6 months pre-COVID; < 6 months pre-COVID; after COVID declared pandemic). Binary logistic regression was used to examine the Independent variables of; “Species” (binary: cat/dog), “Source of pet” (7 categories: Adopted from a shelter/rehoming organization; Purchased from a breeder; As a gift; Directly from someone that needed to find a new home for their cat or dog; Directly from someone that was seeking temporary care for their cat or dog; The cat or dog was found as a stray; Other), “Acquired online” (binary: Yes/No) and “When acquired” (three categories: >6 months pre-COVID; < 6 months pre-COVID; after COVID declared pandemic), on the Dependant Variable = “Giving up pet.” Due to the small number of people that reported having already given their pet up, “Giving up pet” was changed into a binary variable where “I have considered giving up my pet” was combined with “Yes, I have already given up my pet” to form two categories “I have never considered it” vs. “I have considered or have already given up my pet.” Dummy variables were created for any categorical variables with more than two categories. “As a gift” was the reference category for “Source of pet” and “Acquired more than 6 months before COVID-19 was declared a pandemic” was the reference category for “When acquired.” All variables were forced into the model using the function “enter.” SPSS version 25 was used for all analyses.

## 3. Results

### 3.1. Survey 1

**Figure 1:**
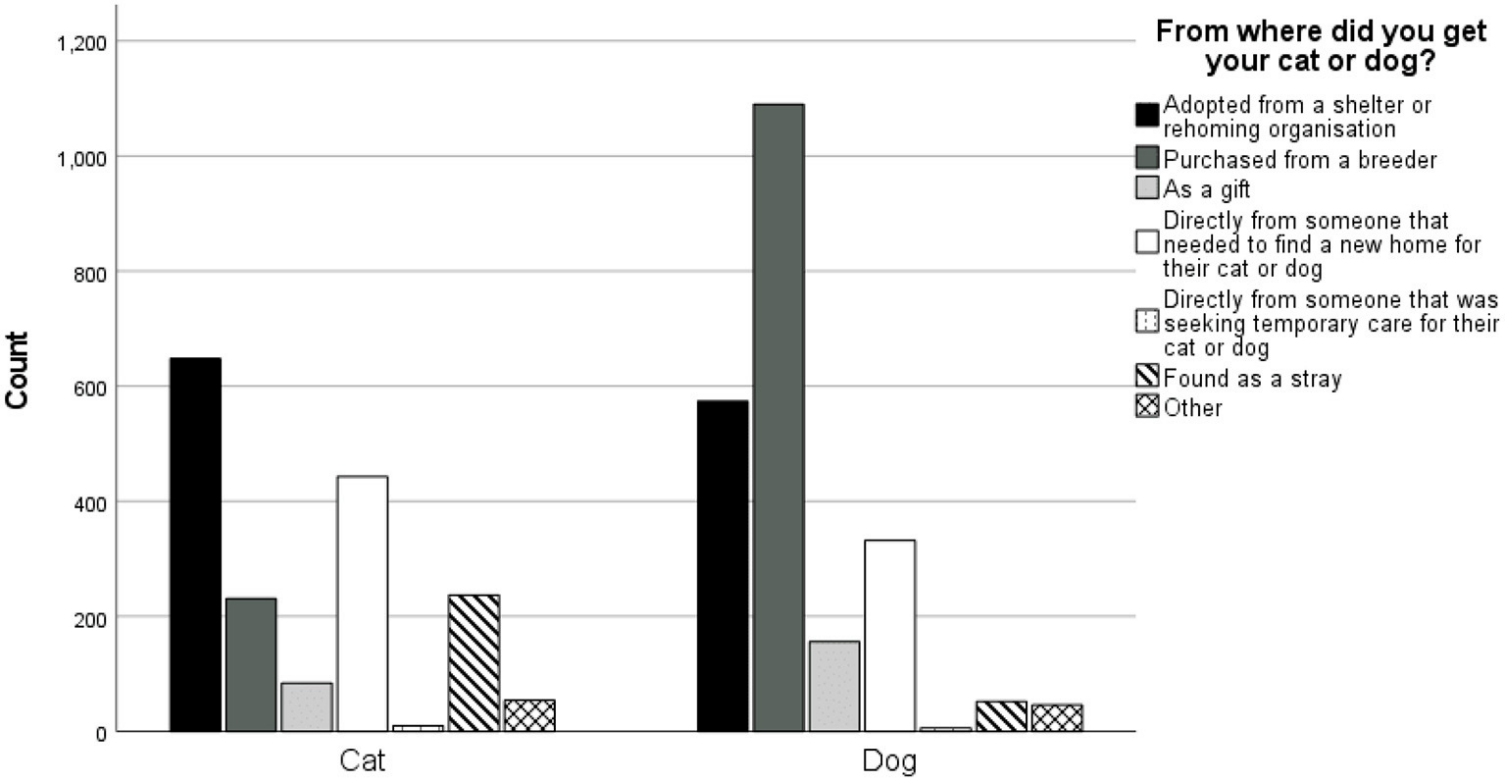
The number of participants acquiring their cats and dogs from each source.

After removal of missing responses, partially incomplete responses and those missing a Prolific ID, the sample size for the screening questionnaire was *N* = 3,945. Participants from 27 countries completed the survey. A total of 58.2% of participants were from the UK (*n* = 2,305), 20.9% from the USA (*n* = 828), 7.6% from Italy (*n* = 301), 4.3% from Canada (*n* = 172), 3.9% from Spain (*n* = 156), 1.5% from Ireland (*n* = 60), 1.2% from Australia (*n* = 47), 1.1% from France (*n* = 42) and 1.3% (*n* = 52) from other countries. Pre-existing demographic information from Prolific Academic was downloaded for each participant. This included age, ethnicity, employment status and student status. Data was missing for a number of participants due to expiration of data on Prolific Academic's system. However, from the available data, the mean age of participants was 34.7(±11.6). In total, 89.8% of participants were white, 2% were black, 3.6% were Asian, 3.6% were Mixed, and 1% selected “other.” A total of 56.8% were in full time employment, 8.8% were in part-time employment, 1.6% were due to start a new job in the next month, 9.7% were not in paid work (e.g., homemaker, retired or disabled), 6.7% were unemployed, and 6.2% reported “other” for employment status. A total of 22.4% of participants stated that they were students. A total of 43.1% of participants completed the survey for a cat while 56.9% completed it for a dog. The number of participants that acquired their cat or dog *via* each source can be seen in Figure 1. Overall, most pets were purchased from a breeder (33.3%) or were adopted from a shelter or rehoming organization (30.8%).

Overall, 85.8% of pet owners obtained their pets more than 6 months before COVID-19 was declared a pandemic, 9% obtained their pets within 6 months before COVID-19 was declared a pandemic and 5.2% obtained their pets after COVID-19 was declared a pandemic. Of those that acquired their pet after COVID-19 was declared a pandemic, 42.7% were not planning on getting this pet beforehand. At the time of survey 1 (11th August 2020), 95.2% of participants had never considered relinquishment (NCR), while 4.2 and 0.5% respectively, had either considered relinquishment (*n* = 168, CR) or had relinquished their pet (*n* = 21, R). A total of 4.0% of cat owners had considered giving up their pet, while 0.5% had already given up their pet. Similarly, 4.4% of dog owners had considered giving up their pet, while 0.5% had already given up their pet.

Of the NCR group (*n* = 3,774), 87.1% acquired their pet more than 6 months before COVID-19 was declared a pandemic, 8.1% within 6 months before COVID-19 was declared a pandemic and 4.8% obtained their pets after COVID-19 was declared a pandemic.

Of the CR group (*n* = 168), 62.5% acquired their pet more than 6 months before COVID-19 was declared a pandemic, 26.2% within 6 months before COVID-19 was declared a pandemic and 11.3% obtained their pets after COVID-19 was declared a pandemic.

Of the R group (*n* = 21), 52.4% acquired their pet more than 6 months before COVID-19 was declared a pandemic, 28.6% within 6 months before COVID-19 was declared a pandemic and 19.0% obtained their pets after COVID-19 was declared a pandemic. Source of companion animal by current ownership status can be found in Table 1. A third of pets (33.7%) were initially found through an online source. In total, 26.6% of cats and 39.0% of dogs were originally sourced online. A 2 (Species: Cat/Dog) X 2 (Online: Yes/No) chi-square analysis revealed this difference to be significant [X^2^ = 67.026 (1), *P* < 0.001].

**Table 1:** Companion animal source by current ownership status^a^.

**Source of pet**	**Never considered relinquishment (NCR) % (*n* = 3,756)**	**Considered relinquishment (CR) % (*n* = 168)**	**Have relinquished (R) % (*n* = 21)**
Adopted from a shelter/rehoming organization	31.2	24.4	19.0
Purchased from a breeder	33.2	35.1	38.1
As a gift	5.5	16.1	19.0
Directly from someone that needed to find a new home for their cat or dog	19.8	15.5	9.5
Directly from someone that was seeking temporary care for their cat or dog	0.3	1.5	14.3
Found as a stray	7.3	7.7	0.0
Other	2.6	0.0	0.0

a We did not receive any free-text responses from those that acquired their pet from someone looking for temporary care. We are therefore unsure of the reason for giving up a pet in this case but it is likely due to only temporary care being needed.

The percentage of pet owners reporting that they acquired their pet online varied according to when their pet was acquired; in total, 31.7% of those that acquired their pet more than 6 months before COVID-19 was declared a pandemic sourced their pet online, compared to 43.9 and 49% of those that acquired their pet within 6 months before, and after COVID-19 was declared a pandemic, respectively. A 2 (Online: Yes/No) X 3 (time acquired: >6 months pre-COVID, < 6 months pre-COVID, after COVID declared pandemic) chi-square analysis revealed this difference to be significant [X^2^ = 44.710 (2), *P* < 0.001]. Bonferroni adjustment was used on all pairwise comparisons (6 groups) using the method outlined by Beasley and Schumacker (52). An adjusted significance threshold of *P* = 0.0083 was set. All values had a *P* < 0.0083, indicating that they were highly statistically significant.

### 3.2. Acquisition characteristics

For the binary logistic regression, the Independent variables: “Species” (binary), “Source of pet” (7 categories), “Acquired online” (binary), and “When acquired” (3 categories) and Dependant Variable: “Giving up pet” were entered into the model. The Hosmer and Lemeshow *P*-value (*P* = 0.704) indicated that the model is a good fit. The logistic regression model was statistically significant, X^2^ (10) = 123.350, *p* < 0.001. Nagelkerke R square value of 0.096 indicated that 9.6% of the variation in the DV is accounted for by the model. There was no effect of species (cat vs. dog) on relinquishment (*P* = 0.835). Compared to pets given as a gift, there was a 38.7% decreased likelihood of relinquishment in pets sourced from shelter (*P* < 0.001), 31.2% decrease in those sourced from a breeder, and a 24.4% decrease in those acquired directly from someone that needed to find a new home for their cat or dog. There was no difference in likelihood of considering or given up a pet in those acquired directly from someone that was seeking temporary care for their cat or dog. Compared to owners who acquired their pet >6 months before COVID-19 was declared a pandemic, those acquired < 6 months before COVID-19 was declared a pandemic were three times more likely to be considered for, or be, given up (*P* > 0.001). Those that were acquired after COVID-19 was declared a pandemic were two times more likely to be considered for, or be, given up (*P* < 0.001). There was a trend for greater likelihood of considering giving up or giving a pet up in those purchased online (*P* = 0.074).

### 3.3. Reasons for relinquishment and methods used to relinquish a cat or dog

Of those that had already given up their pet, three relinquished to a shelter (14.3%), 14 gave their pet to a new owner (66.7%) and four obtained temporary care from someone else (19.0%). Participants had the option of selecting “The cat or dog was let loose.” However, no participants selected this option as a form of relinquishment. Participants were asked for the reason for considering giving up, or giving up, their cat or dog and could select as many answers as desired from 11 options. The most commonly cited reasons for considering giving up, or giving up, their cat or dog are presented in Table 2. Reasons for considering giving up or giving up a pet are broken down by species (cat or dog) and relinquishment status (CR vs. R) in Table 3. Participants were invited to comment further on their reasons if they wished to do so. The most common reasons cited for considering, or given up a pet, were financial constraints. Some participants related their financial problems to the COVID-19 pandemic (e.g., “*Expenses are beyond our budget and I was made unemployed during the lockdown,”* and “*My partner was furloughed since mid March and now is at high risk of being made redundant due to the furlough scheme ending in October”*) while others referred to financial issues more generally that may or may not have been related to the COVID-19 pandemic (e.g., “*Well I would like to give away the second cat I have due to financial reasons,” “Her health issues are expensive”* and “*Concerns about the financial situation”*). Health concerns specific to COVID-19 were also frequently selected as a reason for considering or having already relinquished a pet, being the second most frequently cited reason (e.g., “*I was scared that the Dog might contract COVID-19 especially when I heard that animals have likelihood of getting the disease”* and “*the fear of inadequate information on the transmission of COVID 19 is an issue for me”*). Behavioral concerns were the third most commonly cited reason for considering or having already relinquished a pet (e.g., “*My new puppy has developed into a car chaser. This is proving a huge problem and despite intensive training from a professional, it is getting worse”* and “*He's a handful”*).

**Table 2:** The reasons cited for considering, or actually given up a pet, in order of overall frequency.

Reason for considering or actually giving up a pet^*^	**Overall % (*n* = 189)**
1) Financial constraints have made it difficult to care for my cat or dog	44.2
2) Health concerns specific to COVID-19 (e.g., fear of cat or dog transmitting the virus to yourself or family members)	32.6
3) Behavioral concerns (e.g., house soiling, barking)	30.9
4) Safety concerns (e.g., the animal is aggressive to myself or others)	21.5
5) I feel as though I have not had enough time to properly care for the cat or dog	17.7
6) Personal reasons (e.g., divorce, my partner does not like the animal)	16.6
7) Health concerns (e.g., allergies)	16.6
8) I have increased work hours due to being an essential worker during the COVID-19 pandemic	16.6
9) The cat or dog does not get along with other pets in the household	9.4
10) I have moved house and could not bring my cat or dog	8.3
11) Other	7.2

* Participants could select as many reasons as desired.

**Table 3:** The reasons cited for giving up a pet by species (cat or dog) and consideration of relinquishment compared to actual relinquishment.

Reason for considering or actually giving up a pet^*^	**Cat % (*n* = 78)**	**Dog % (*n* = 101)**	**Considered relinquishment (CR) % (*n* = 168)**	**Have relinquished (R) % (*n* = 21)**
Financial constraints have made it difficult to care for my cat or dog	44.7	43.8	45.0	38.1
Health concerns specific to COVID-19 (e.g., fear of cat or dog transmitting the virus to yourself or family members)	19.7	41.9	34.4	19.0
Behavioral concerns (e.g., house soiling, barking)	31.6	30.5	30.0	38.1
Safety concerns (e.g., the animal is aggressive to myself or others)	19.7	22.9	20.0	33.3
I feel as though I have not had enough time to properly care for the cat or dog	18.4	17.1	17.5	19.0
Personal reasons (e.g., divorce, my partner does not like the animal)	21.1	13.3	14.4	33.3
Health concerns (e.g., allergies)	17.1	16.2	15	28.6
I have increased work hours due to being an essential worker during the COVID-19 pandemic	17.1	16.2	11.3	23.8
The cat or dog does not get along with other pets in the household	7.9	10.5	8.1	19.0
I have moved house and could not bring my cat or dog	6.6	9.5	7.5	14.3
Other	13.2	2.9	7.5	4.8

* Participants could select as many reasons as desired.

### 3.4. Follow-up surveys

#### 3.4.1. Further relinquishment

Those that reported having considered giving up or having already giving up their pet were invited to take part in a second survey 1 week later (*n* = 189). Data was collected between 17th and 24th of August, 2020. Of those invited, *n* = 153 usable responses were received. Five participants gave up their pet in the week between survey 1 and survey 2, moving from considering giving up their pet (CR) to actual relinquishment (R). Seven months later, participants that had competed the survey 2 were asked if they would like to participant in a revisit. Data was collected between 31st March and 8th April 2021. Of those invited, responses were received from a total of *n* = 64 participants, with no response from the remaining 104 individuals that were considering relinquishment (CR) when completing survey 2. Of these, two pets passed away, one was given to a shelter or rehoming organization, and two were given away directly to a new owner. Therefore, three participants gave up their pet over the 7 months between survey 2 and survey 3.

#### 3.4.2. Gender

In total, 65.8% of those considering or having already relinquished their pets were male, 33.5% were female, 0.6% preferred not to say, and 0% identified as non-binary. When broken down, 65.0% of those considering relinquishment were male, increasing to 72.2% of those that have already given up their pets.

## 4. Discussion

### 4.1. Relinquishment of cats and dogs during the COVID-19 pandemic

In this study, we explored adoption and relinquishment of companion animals during the COVID-19 pandemic. Overall, considering data from the three surveys, 4.06% of participants considered giving up their pet (168/3945), 0.74% (28/3945) gave up their pet, and 0.2% considered and then did give up their pet (8/3945). This suggests that most pet owners that participated in this research have not considered relinquishing their pets during the time period examined. While five participants reported having given up their pet in the week between survey 1 and survey 2, only three more reported moving from consideration of relinquishment to actual relinquishment in between survey 2 and survey 3. However, only a sub-sample of participants responded to survey three. Therefore, the retention rate for all participants considering relinquishment could not be established. In any case, the relatively low percentage of CR participants that eventually relinquished pets suggests that most individuals are reluctant to give up their pets. This supports evidence from Dolan et al. (16) that a majority of pet owners are willing to consider options other than relinquishment. This suggests that there is time and opportunity to intervene in order to avoid companion animal relinquishment. However, it should be noted that most participants were white, in full time employment and largely from the UK and USA. Furthermore, those that use the Prolific Academic website to complete surveys tend to be white, have high English fluency, a medium income level, and have a third level qualification (30). Therefore, these results may not be generalizable to pet owners more generally and should be treated with caution.

While still relatively low, the number of pet owners considering relinquishment in the current study is higher than that of other studies. For example, Morgan et al. (23) reported that 2.6% of Israeli dog owners returned, or considered returning, their dogs to shelters during the COVID-19 pandemic. Brand et al. (31) examined puppy acquisition in a large sample of UK puppy owners (*N* = 5,517) and also reported a lower level of relinquishment at 0.9% for puppies acquired in 2019 and 1.2% for those acquired in 2020. However, these studies focused on relinquishment to shelters only. Duarte Cardoso et al. (27) assessed relinquishment of cats and dogs in Portugal that included both illegal abandonment and relinquishment to a shelter and found that 3.43% of respondents reported to have relinquished a cat or dog in the past (36/1,049), a figure more in line with our findings. In the current study, most pets that were relinquished (66.7%) were given directly to a new owner. Similarly, Hoffman et al. (32) found that, of cats and dogs acquired during the COVID-19 pandemic that were no longer with their owners, 50% of dogs and 36.8% of cats had been given to a friend, family member or neighbor. This could suggest that shelter data may under-estimate the number of individuals that give up their companion animals. In the current study, no participants selected “The cat or dog was let loose” as a means of giving up their pet. However, this may be due to social desirability bias which can occur in research that depends on owners self-reporting their experiences (33, 34). Further research is needed to identify risk factors for surrender of pets *via* a variety of means. It would also be of interest to identify the ultimate fate of animals relinquished to shelters compared to friends, family members and other third parties. However, a greater sample of relinquishers is needed.

### 4.2. Companion animal source

There was no effect of species (cat or dog) on risk of relinquishment. There were however, differences between cats and dogs in terms of reasons for relinquishment. These are discussed below in Section 4.5. Where the animal was sourced was related to relinquishment. Individuals that had already given up their pet were more likely to have been given the animal as a gift or directly from someone that was seeking temporary care for their cat or dog, compared to pet owners that had never considered giving up their pet. In previous studies, the source of pet has been classified according to the pet-owners' intentions on acquisition. For example, Zito et al. (35) classified acquiring a cat as a gift, or being left with the surrender by another person, as passive methods of pet acquisition. Those acquired from animal shelters or breeders were considered to have been actively acquired. Similar to Zito et al. (35), Holland et al. (36) classified those given as a gift, or dogs in need of a home, as unplanned, while those acquired in a deliberate search for a pet were classified as planned. While the proportion of participants reporting to be considering giving up or having already given up their pet in the current study was small, the intention to acquire a pet, or lack thereof, may help to explain the current study findings. Indeed, compared to those acquired as a gift, those acquired through “active” or “planned” routes (sourced from a shelter, breeder or private transaction) were at decreased risk of relinquishment. Interestingly, animals found as a stray were not at increased risk of relinquishment, despite the unplanned or passive manner of this type of companion animal acquisition. It is possible that pet owners may be less invested or attached to pets that they may have felt obliged to provide care for. Indeed, Holland et al. (36) interviewed dog owners about their motivation for pet acquisition and found that unplanned acquisitions were often from family or friends, with some pet owners actively volunteering to help, willing to help when asked, or feeling that they had little choice in the matter. Perception of choice in unexpected pet acquisition is likely important in determining future relinquishment of such pets. The increased risk of relinquishment for pets obtained as a gift is in line often voiced concern from animal charities and organizations that animals given as gifts may end up in shelters (37–39). However, this finding contrasts with most previous research, where pets given as a gift were not found to be at increased risk of relinquishment (40). For example, Scarlett et al. (41) found that only 0.3% of dog owners and 0.4% of cat owners cited “unwanted gift” as a reason for relinquishment across 12 USA animal shelters. Similarly, New et al. (29) found that very few animals (2.9%) relinquished across 12 USA animal shelters were originally given to the owner as a gift. However, Scarlett et al. (41) and New et al. (29) appear to have used the same dataset of 12 USA shelters in their studies. Furthermore, these studies were conducted approximately 20 years ago and may not be an accurate reflection of pet acquisition and relinquishment today. More recently, Weiss et al. (42) assessed relinquishment of, and attachment to, pets given as gifts. Weiss et al. (42) made a distinction between different levels of involvement in the gifting process from the pet being a surprise to the owner being involved in selecting the gifted pet, and found that gifted animals, regardless of owner involvement in the process, were not at increased risk of relinquishment. Furthermore, owners of gifted pets were just as attached to their animals as other pet owners were. A distinction between surprise gifts vs. those that were expected was not made in the current study. Montoya et al. (17) assessed the effect of the source of pet acquisition on later euthanasia risk in one Australian animal shelter and found that both adult cats and dogs were at increased risk of euthanasia if they were originally acquired as gifts. However, a “gift” included any animal acquired at no cost from family and friends. Therefore, it is impossible to determine how many of these animals were truly given as gifts and how many were actively sought out by the pet owner. Given the current study findings, more research is needed to assess the risk of relinquishment in pets acquired as gifts, with a distinction being made between unexpected gifts and those where the pet owner was actively involved in the acquisition process.

#### 4.2.1. Online sourcing of companion animals

Overall, one third of pets in the current study were acquired *via* online means, with dogs being acquired online significantly more often than cats. There was an increase in the number of pets being purchased online over time; a third of pets acquired more than 6 months before COVID-19 was declared a pandemic were acquired online, increasing to almost 50% in those acquired post-pandemic. During the COVID-19 pandemic, Google searches for puppies increased in several countries (19) and it appears that this increase in searches has indeed been converted into increased pet acquisition. Increases in online acquisition post-pandemic may be due to shelter closures and related lockdown restrictions. However, the increase in online acquisition in the months preceding this suggests an overall increase in online sourcing of pets more generally. Similar to the current study, Packer et al. (28) found that 34.5% of 2019 puppies and 45% of 2020 puppies were acquired *via* selling websites. In terms of risk of relinquishment, there was no effect of online acquisition on relinquishment behavior (*P* = 0.074) in the current study. However, given the small sample size of those reporting to have already given up a pet, further research is needed as numerically, there was a higher prevalence of consideration of relinquishment of pets acquired online (5.8%) compared to other means (4.2%). While the increased acquisition of pets *via* online means is concerning, the internet also allows opportunity for intervention, such as regulation and monitoring (20) which should be explored in future research.

### 4.3. Time of acquisition

Time of companion animal acquisition was related to relinquishment. In a similar study. Hoffman et al. (32) classified pet acquisition slightly differently but uncovered comparable findings; compared to USA pet owners that did not acquire a new pet during the pandemic, those that got new pets post-pandemic were between 3 and 5 times more likely to consider relinquishment, and between 3 and 7 times more likely to actually relinquish a pet during the pandemic. However, Hoffman et al. (32) did not assess whether the relinquished pets were acquired during the pandemic or not. In the current study, it was animals that were acquired within 6 months before COVID-19 was declared a pandemic that were at greatest risk of relinquishment. Together, these results suggest that long-term pet owners were are less likely to relinquish or consider relinquishing pets during the pandemic. The reduced risk of relinquishment in those acquiring their pet post-pandemic compared to < 6 months pre-pandemic may be because individuals that decided to get a pet at this time have considered the impact of lockdowns and related lifestyle changes when making their decision to acquire a pet. For example, Packer et al. (28) found that, compared to 2019, puppies were acquired more often during 2020 that suited the owner's lifestyle, were good with children, were easy to train and encouraged exercise. These decisions may have been related to the increased number of people working from home, home schooling, and restrictions on leaving the home for non-essential reasons (32, 43–45). The COVID-19 pandemic also afforded new pet owners more time to train new pets (11, 32). In contrast, owners of relatively new pets that were acquired before the COVID-19 pandemic were thrust into a new pet-ownership relationship unexpectedly, with a pet that may not have characteristics suited to the new post-pandemic environment. For example, existing pet owners may need to make adjustments to how they look after their pets such as re-training cats or dogs to toilet indoors (3). Overall, our findings suggest that it is owners of new pets that require the most support in terms of avoiding relinquishment. Those that acquired a new companion animal prior to the COVID-19 pandemic may particularly need support in helping them to adjust to the ever-evolving situation.

### 4.4. Pet-owner gender

A strength of the current study is the balance of male and female participants that were recruited. Despite male/female imbalances in previous studies, differences in relinquishment rates between males and females have been uncovered. For example, New et al. (29) examined the demographics of pet owners who relinquished their pets at 12 USA shelters and compared them to the demographics of a sample of USA households that contained pets. New et al. (29) found that 50.5% of dogs were relinquished by males, while 24.9% of current dog owners were male, and 38.9% of cat relinquishers were male, while 20.4% of cat owners were male. This suggests that males were more likely to relinquish animals to a shelter relative to the number that own cats and dogs. More recently, Dolan et al. (16) used a similar approach, were companion animal-relinquisher demographics were contrasted with a comparison group of pet-owners who were at the shelter to avail of low-cost/free neutering service. Dolan et al. (16) found that 38.5% of relinquishers were male, compared to 20.8% of participants in the comparison group. However, it is important to note that both of these studies grouped participants based on owner sex rather than gender. In line with our work and use of gender, Hoffman et al. (32) analyzed data on animal acquisition and relinquishment that had an even split between self-identifying males and females (47% male) and found that USA males were more likely to have acquired a pet, considered relinquishment and actually relinquished pets during the pandemic, compared to females. Our findings strengthen the evidence that males are at increased risk of relinquishing cats and dogs; 65% of those considering giving up a pet, and 72.2% of those having already given up a pet, identified as male. Future interventions to address pet relinquishment should be designed with this difference in mind. There are also other gender imbalances that need to be addressed in future work. The current work, and much of prior work, uses predominantly male and female gendered or sexed participants, meaning results may not be generalizable to the whole population. It is important that further research is conducted with even gender splits among other genders, such as non-binary, to move toward a more accurate and representative overview of pet ownership and relinquishment.

### 4.5. Reasons for relinquishing a pet

Financial constraints were the most mentioned reason for both giving up a pet and considering giving up a pet. Pet owners often under-estimate the financial burden associated with pet ownership (36) and companion animals may compound existing stressors in households in financial difficulty (45). For example, Eagan et al. (40) found that 10% of dog owners cited financial reasons as the reason for relinquishment of dogs to a number of Canadian shelters between 2008 and 2019. Similarly, Applebaum et al. (4) distributed an online survey to a convenience sample of 3006 USA pet owners, and found that 7% of participants reported concerns and difficulties related to financial problems and caring for their pets during the COVID-19 pandemic. In the current study, participants could select as many reasons for relinquishment as desired from a set of eleven and this may explain the increased number of participants citing financial reasons compared to previous studies. For example, finances may be related to other relinquishment reasons rather than being the primary reason in all cases. Future research should include a ranking of reasons to elucidate the most important influences on the decision to relinquish a pet. Country of residence may influence the importance of financial constraints on companion animal relinquishment. Detailed information on relinquishment by country are discussed elsewhere (Carroll et al. in preparation).

The second most cited reason for considering or relinquishing a pet was health concerns specific to COVID-19. The example given to participants for this option was “e.g., fear of cat or dog transmitting the virus to yourself or family members.” The free-text responses received support the idea that pet owners fear that their pets could contract the virus and pass it to humans. Two interesting findings in relation to this were that; (1) Dog owners cited this concern more often than cat owners, and (2) participants cited this concern as a reason for considering relinquishment more often than as a reason for actual relinquishment. The first case of potential pet to human transmission was reported in a dog in Hong Kong. This is purported to have instigated a number of pet killings to avoid spreading the virus (25). However, evidence suggests that the chance of companion animals spreading COVID-19 is low (43). Indeed, COVID-19 is more likely to be transmitted from humans to their pets than vice-versa (26, 46). While there is evidence that transmission from mink to humans has occurred, most evidence shows unidirectional transition of COVID-19 from humans to several animal species including cats and dogs (47). However, this does not appear to have quelled fear in members of the public. Data collection for the current study began in August 2020, just 5 months after COVID-19 was declared a pandemic. Fears of pet to human transmission appears to have had a big impact in this time when fear and unfamiliarly with the virus was prevalent. Interestingly, it was cats that were identified by researchers as potential hosts of the virus rather than dogs (48, 49). Despite this, many more dog owners cited health concerns specific to COVID-19 as a reason for considering or relinquishing a pet. Cats can be kept indoors more easily than dogs who require exercise outside of the home (2). This may explain the difference in concerns between cat and dog owners as dogs may be difficult to keep away from members of the public who may be infected with COVID-19. More research is needed to assess differences in fear of transmission of COVID-19 between cats, dogs and their owners. Jensen et al. (50) highlight the need to distinguish between real and perceived problems. Given the lack of evidence of pet to human transmission of the virus, the risk of pet to human transition of COVID-19 is an example of a perceived problem and highlights the importance of pet owner perceptions in the relinquishment process. The public should be better educated about the true risk of transmission of COVID-19 to and from companion animals to avoid unnecessary companion animal relinquishment.

The third most cited reason for relinquishment was behavioral concerns. The importance of behavioral problems on relinquishment varies across studies and species. For example, Jensen et al. (50) assessed longitudinal data from one Danish shelter and found that behavioral problems were the most cited reason for relinquishment of dogs but were less important in cat relinquishment. In a systematic review of reasons for surrendering dogs to shelters, Lambert et al. (14) found that behavioral problems were cited as a reason in 10.8–34.2% of studies from around the globe. In the current study, behavioral problems were reported at similar levels for both species under investigation. Behavioral problems may become more apparent during lockdowns as owners have more time to notice issues. Additionally, the nature of lockdowns themselves, i.e., working from home, home schooling and limited time permitted outdoors, can negatively affect animal behavior, both at the time and into the future as restrictions ease. For example, more time spent at home may increase the risk of aggression toward children (51). Indeed, Tulloch et al. (44) found that the incidence of dog bites increased at pediatric emergency departments during UK lockdown periods. Furthermore, Brand et al. (31) found that puppies purchased during the pandemic were left alone less often and had less experience of public spaces, and humans and dogs outside of the family unit. Poor socialization can, in turn, increase the risk of aggression in dogs in the future (31). What's more, problems may be exacerbated by inexperience with pet ownership in those that acquired a pet post-pandemic (43). The current study findings suggest that behavioral concerns remain as a key consideration in companion animal relinquishment. Jensen et al. (50) highlight the importance of distinguishing between owner-related and pet-related reasons for relinquishment as they may require varying solutions. In the current study, the main reasons for considering relinquishment, or actually relinquishing a pet, were for financial, health concerns related to COVID-19, and behavioral problems for dogs. This suggests that interventions that target both animal behavior and human expectations are required.

## 5. Conclusion

The concern around relinquishment of companion animals in recent times has focused on those acquired during the pandemic. Our findings suggest that cats and dogs acquired post-pandemic are indeed at increased risk of relinquishment compared to those acquired over 6 months beforehand. However, it was those acquired in the 6 months before the COVID-19 pandemic that were at particular risk of relinquishment. Greater support is needed for this group of pet owners. This study provides strong evidence that males are at increased risk of relinquishing pets compared to females. The reasons for this difference should be explored further and relinquishment interventions should be designed with this gender difference in mind. Most individuals that relinquished a pet, gave the animal away to a new owner. More research is needed into this type of relinquishment to establish the true prevalence of companion animal relinquishment, and to establish the ultimate outcome of pets relinquished in this manner. Fear of pet to human transmission of COVID-19 is playing a role in companion animal relinquishment, despite the lack of evidence of pet to human transmission of the virus. Greater education of members of the public on this topic is required. There appears to be a shift toward online acquisition of pets in recent times. More research needed on effect of online acquisition on relinquishment of cats and dogs. Contrary to previous research, pets acquired as gifts were found to be at increased risk of relinquishment. More research on this is needed, given the conflicting findings within the literature. Finally, less than 10% of the variation in relinquishment intention was explained by the factors assessed in the current study. This suggests that other factors are also at play. Owner demographics, such as home ownership status and family composition, will be explored in a separate publication as part of the CAARP project.

## Data availability statement

The raw data supporting the conclusions of this article will be made available by the authors, without undue reservation.

## Ethics statement

The studies involving human participants were reviewed and approved by Queen's University Belfast Faculty Research Ethics Committee (EPS 20_111). The patients/participants provided their written informed consent to participate in this study.

## Author contributions

GC and CR contributed to conception and design of the study and acquired the funding for this research. GC and AT performed the data analyses. GC wrote the first draft of the manuscript and provided supervision. AT wrote sections of the manuscript. All authors contributed to manuscript revision, read, and approved the submitted version.

## Funding

This research was funded by the Society of Companion Animal Studies (SCAS). Grant reference code: PPSCAS1004.

## Conflict of interest

The authors declare that the research was conducted in the absence of any commercial or financial relationships that could be construed as a potential conflict of interest.

## Publisher's note

All claims expressed in this article are solely those of the authors and do not necessarily represent those of their affiliated organizations, or those of the publisher, the editors and the reviewers. Any product that may be evaluated in this article, or claim that may be made by its manufacturer, is not guaranteed or endorsed by the publisher.
